# Analysis of MRPL23 protein expression and its role in prostate cancer pathogenesis

**DOI:** 10.1093/carcin/bgaf078

**Published:** 2025-12-20

**Authors:** Edyta Podemska, Damian Łukasik, Jędrzej Borowczak, Dariusz Grzanka, Justyna Durślewicz

**Affiliations:** Department of Clinical Pathomorphology, Faculty of Medicine, Collegium Medicum in Bydgoszcz, Nicolaus Copernicus University in Torun, 85-094 Bydgoszcz, Poland; Department of Clinical Pathomorphology, Faculty of Medicine, Collegium Medicum in Bydgoszcz, Nicolaus Copernicus University in Torun, 85-094 Bydgoszcz, Poland; Clinical Department of Oncology, Franciszek Łukaszczyk Oncology Centre, 85-796 Bydgoszcz, Poland; Medical Faculty of University of Science and Technology in Bydgoszcz, 85-796 Bydgoszcz, Poland; Department of Clinical Pathomorphology, Faculty of Medicine, Collegium Medicum in Bydgoszcz, Nicolaus Copernicus University in Torun, 85-094 Bydgoszcz, Poland; Department of Clinical Pathomorphology, Faculty of Medicine, Collegium Medicum in Bydgoszcz, Nicolaus Copernicus University in Torun, 85-094 Bydgoszcz, Poland; Medical Faculty of University of Science and Technology in Bydgoszcz, 85-796 Bydgoszcz, Poland; Department of Tumor Pathology and Pathomorphology, Prof. Franciszek Łukaszczyk Oncology Centre, 85-796 Bydgoszcz, Poland

**Keywords:** prostate cancer, MRPL23, immunohistochemistry, patient survival, prognostic biomarker

## Abstract

Prostate cancer (PCa) is the fourth most commonly diagnosed malignancy worldwide and remains a major clinical challenge due to its heterogeneous course and lack of reliable prognostic biomarkers. Mitochondrial ribosomal protein L23 (MRPL23) has recently emerged as a potential contributor to cancer progression, but its role in prostate cancer remains poorly understood. Formalin-fixed, paraffin-embedded (FFPE) tissue samples from 67 PCa patients who underwent radical prostatectomy were analyzed. MRPL23 expression was assessed by immunohistochemistry using a semi-quantitative immunoreactive scale (IRS). Clinicopathological data were collected for correlation analysis. Survival outcomes were evaluated using Kaplan–Meier curves and Cox proportional hazards models. MRPL23 expression differed significantly across all tissue types, with higher levels in prostate cancer tissues compared with normal epithelium, and the highest expression observed in lymph node metastases (*P* < .001). High MRPL23 expression was associated with shorter overall survival (*P* = .003) and remained an independent prognostic factor in the multivariate analysis (HR 3.99, 95% CI 1.63–9.77, *P* = .002). Complementary TCGA analysis confirmed elevated MRPL23 mRNA levels in prostate adenocarcinomas compared with normal tissues (*P* = .01) and demonstrated that high expression predicted shorter disease-free survival (10-year DFS: 75.98% versus 92.92%, log-rank *P* = .01). MRPL23 is a potential prognostic biomarker in prostate cancer, linked to aggressive tumor behavior and poor outcomes. Its expression in metastatic tissue suggests a role in disease progression, while TCGA data confirm its prognostic value for recurrence risk. MRPL23 may also serve as a therapeutic target in advanced PCa.

## Graphical Abstract

**Graphical Abstract:**
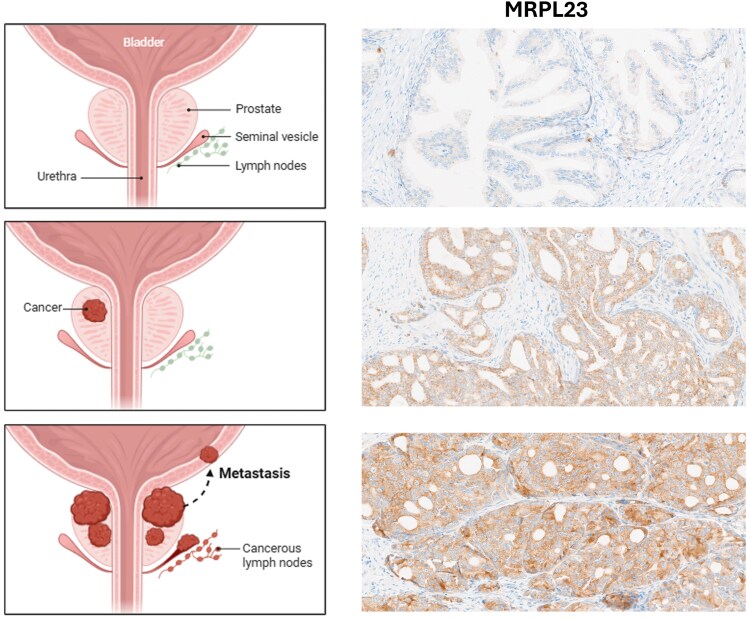

## 1. Introduction

Prostate cancer (PCa) is the fourth most commonly diagnosed malignancy worldwide, with over 1.41 million new cases in 2020. In 2023, PCa accounted for nearly 288 300 new cases and 34 700 deaths, making it the second leading cause of cancer-related deaths in U.S. men. The global burden of PCa is steadily increasing, with 2.43 million new cases and 740 000 deaths projected by 2040, primarily due to population growth and aging [1]. Despite advances in understanding and treatment, the global burden of prostate cancer continues to grow, with rising incidence and mortality highlighting the need for further research.

Over 95% of malignant tumors arising in the prostate are adenocarcinomas. Other histological subtypes, such as neuroendocrine or urothelial carcinoma, are rare and exhibit distinct biological behavior. The risk of developing PCa increases with age and is strongly associated with somatic or hereditary genetic mutations, including mutations in the BRCA2 or HOXB13 genes. While ∼90% of all prostate cancers are detected at a localized stage, 10% of patients are diagnosed with advanced, often metastatic disease. In such cases, the 5-year relative survival rate falls drastically [2–4]. Despite advances in clinical management, biomarkers for reliable risk stratification and individualized treatment of prostate cancer are still lacking.

Mitochondrial ribosomal proteins (MRPs) have recently gained attention as potential molecular biomarkers involved in cancer progression [5–9]. They participate in mitochondrial translation, playing a critical role in protein synthesis, particularly of subunits involved in oxidative phosphorylation (OXPHOS) [10]. The MRP family consists of ∼80 genes, categorized into those forming the large (MRPL) and the small (MRPS) subunits of the ribosome, respectively [11, 12]. Changes in MRP expression can alter reactive oxygen species (ROS) levels and downstream pathways regulating apoptosis, proliferation, and genomic stability [13, 14].

Within this protein family, MRPL23 has emerged as a potentially important, though poorly characterized, factor in oncogenesis. While little is known about its role in prostate cancer specifically, data from other malignancies point to its upregulation and functional relevance [15]. Disruption of mitochondrial translation can lead to impaired bioenergetics and a metabolic shift toward aerobic glycolysis (Warburg effect), which favors tumor growth in hypoxic conditions [12, 15–17]. TCGA data suggest a link between MRPL23 expression and tumor phenotype, supporting its further investigation as a potential diagnostic and prognostic biomarker in prostate cancer.

This study aims to evaluate the expression of MRPL23 and its association with prostate cancer progression and patient survival. Since previous research has demonstrated the involvement of MRPL23 in the initiation and development of other tumors, analogous mechanisms may also contribute to prostate cancer biology. Given the limited understanding of MRPL23 in this context, elucidating its function may help identify new prognostic biomarkers and support the development of targeted therapies.

## 2. Materials and methods

### 2.1. Patient characteristics and tissue material

The study cohort included archival formalin-fixed, paraffin-embedded (FFPE) samples collected at the Department of Clinical Pathology between 2016 and 2019 from 67 prostate cancer patients, who underwent radical prostatectomy at the General Urology and Oncology Clinic in Bydgoszcz. Clinicopathological data of patients—i.e. age, stage T (pT), stage N (pN), distant metastasis (M), grading, Gleason score, and PSA level were obtained using the hospital IT system with electronic patient records. Tumor characteristics were standardized according to the 8th edition of the American Joint Committee on Cancer (AJCC) [18]. Detailed patient and tumor data are provided in Supplementary Table S1. The study group included 67 prostate cancer tissue samples, while the control sample consisted of 30 normal (adjacent non-cancerous) tissues. Among the 23 patients classified as pN1, lymph node metastasis FFPE samples were available in 18 cases, and these were included in the analysis. Overall survival (OS) was defined as the time from the date of diagnosis to death or last follow-up. The median OS was 1787 days, with follow-up ranging from 9 to 2576 days. The study was approved by the Ethics Committee of the Nicolaus Copernicus University in Toruń, Ludwik Rydygier Collegium Medicum in Bydgoszcz (KB 248/2019).

### 2.2. Tissue macroarrays and immunohistochemical staining

Tissue macroarrays (TMAs) were prepared from FFPE tissue samples from selected tumor areas containing at least 80% tumor cells. Each recipient block contained 15 tissue cores, 4 mm in diameter, three of which came from the same patient. Control arrays were created from histologically normal tissue adjacent to the tumor and consisted of one tissue core from each of 30 patients in the study cohort. For each immunohistochemical (IHC) assays, the TMA block were cut into 4-μm-thick sections using a manual rotary microtome (Accu-Cut, Sakura, Torrance, CA, USA) and mounted on adhesion-enhanced slides (Superfrost Plus; Menzel-Glaser, Braunschweig, Germany). The slides were pre-deparaffinized at 60°C for 1 h.

IHC staining was performed as previously described, following a standardized protocol using the BenchMark® ULTRA automated slide processing system (Ventana Medical Systems, Tucson, AZ, USA) [19]. Detailed information regarding the antibodies, incubation times, and detection conditions is provided in Supplementary Table S2. After staining, the slides were dehydrated through a graded ethanol series and cleared in xylene. Finally, they were mounted using a coverslip and Shandon Consul-Mount medium (Thermo Scientific, Waltham, MA, USA). A positive control was selected based on information from the manufacturer's datasheets and the Human Protein Atlas (http://www.proteinatlas.org).

### 2.3. Immunostaining evaluation

The assessment of protein expression in IHC-stained slides was performed on tumor tissues, control group tissues, and lymph node metastases. The slides were scanned using a Roche Ventana DP 200 digital scanner and independently evaluated by both an image scientist and a pathologist, blinded to clinical data. Inter-observer reproducibility was assessed using Cohen’s *κ* coefficient (*κ* = 0.94). Discrepancies occurred in 6 cases (5.2%) and were resolved through joint discussion; when consensus could not be reached, the final decision was made by the senior pathologist. Immunoexpression analysis was carried out using a modified Remmele–Stegner immunoreactive scale (IRS). This semi-quantitative scale ranges from 0 to 12 and takes into account both the percentage of positively stained cells (scored 0–4) and the intensity of staining (scored 0–3).

IHC results for MRPL23 were classified according to the percentage of positive tumor cells as follows: 0 (no positive cells), 1 (<10%), 2 (10%–50%), 3 (51%–80%), 4 (>80%). Staining intensity was scored as 0 (negative), 1 (weak), 2 (moderate), and 3 (strong). Based on the IRS score, protein expression levels were divided into low-expression and high-expression groups using the Evaluate Cutpoints program to determine the optimal threshold [20].

### 2.4. In silico analysis

Clinical data from The Cancer Genome Atlas (TCGA) Prostate Adenocarcinoma (PRAD) cohort were retrieved via the cBioPortal platform [21]. Fragments per kilobase of transcript per million (FPKM) gene expression data were obtained from the University of California, Santa Cruz (UCSC) Xena browser (TCGA PRAD dataset) [22]. Since MRPL23 expression in both tumors and matched normal tissues followed a normal distribution and homogeneity of variances was confirmed, mean mRNA levels were compared using Student’s *t*-test. For the purpose of survival analysis and stratification, MRPL23 expression was dichotomized using the Cutoff Finder online tool, and the cutoff was set at FPKM = 3339 [23]. The Cox models were tested using the proportional hazard test and the Schoenfeld’s rests were analyzed.

### 2.5. Statistical analysis

Statistical analysis was performed using GraphPad Prism version 10.01 (GraphPad Software, La Jolla, CA, USA) and SPSS version 29.0 (IBM Corporation, Armonk, NY, USA). The normality of data distribution was assessed using the Shapiro–Wilk test. Continuous variables were compared using the Mann–Whitney test, and the significance of clinical factors was evaluated using the chi-square test or Fisher’s exact test. Survival analysis was conducted using the Kaplan–Meier method, and differences between groups were assessed using the log-rank test. Univariate and multivariate Cox proportional hazards regression analyses were performed, adjusting for clinical factors such as age, Gleason group, pT, pN and PSA, to estimate hazard ratios (HRs) and corresponding 95% confidence intervals (CIs). A *P*-value <0.05 was considered statistically significant.

## 3. Results

### 3.1. The association between MRPL23 expression and clinicopathological features

**Figure 1.**
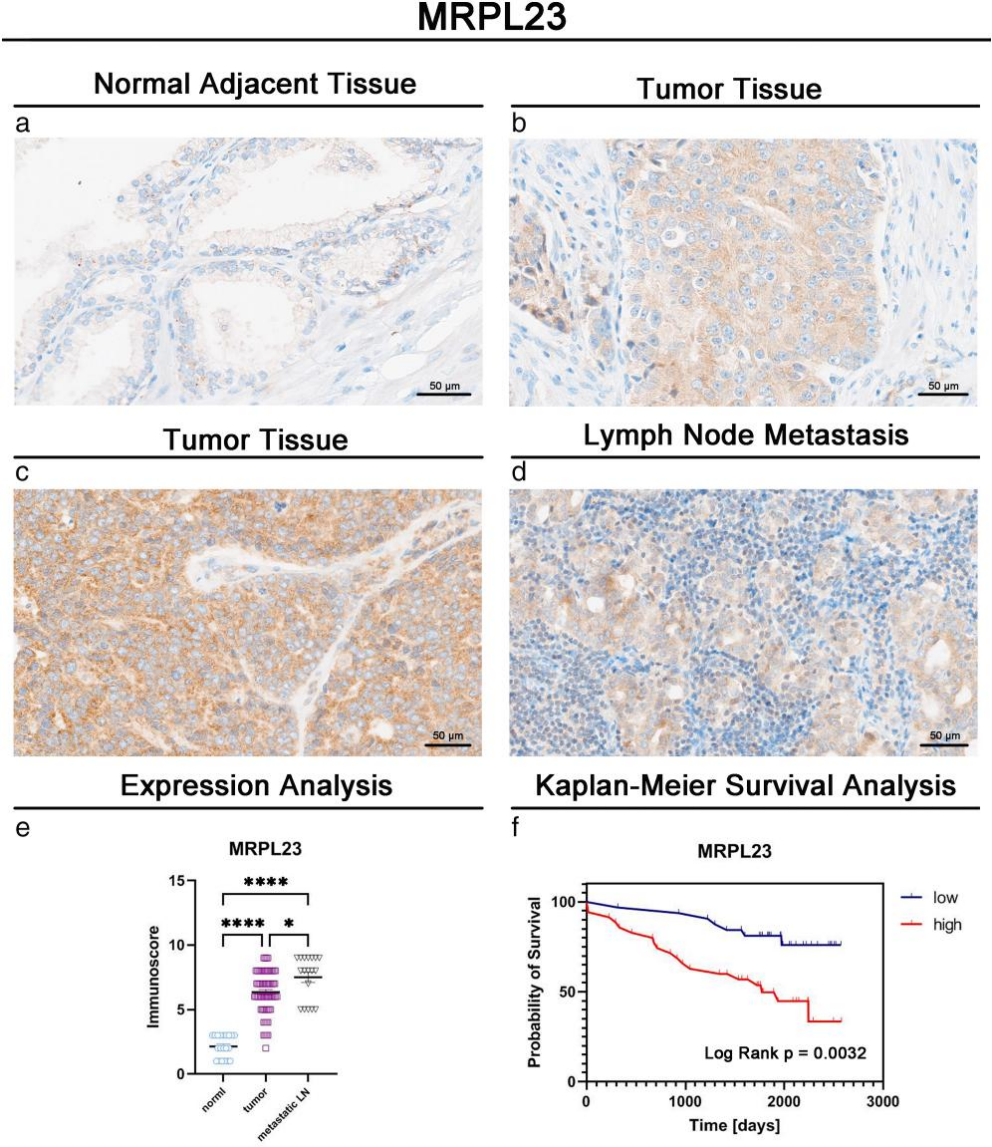
Immunohistochemical and statistical analysis of MRPL23 in PCa patients. (a–d) Representative immunohistochemical staining of MRPL23 in prostate cancer tissues. (e) Comparison of MRPL23 expression levels in normal tissues, primary tumors, and lymph node metastases. (f) Kaplan–Meier survival curves with log-rank test results for overall survival according to MRPL23 expression status

IHC staining of MRPL23 was observed in the cytoplasm of normal tissues, primary tumors, and lymph node metastases. A similar cytoplasmic staining pattern in PCa samples was also documented in the Human Protein Atlas (http://www.proteinatlas.org). Representative staining patterns in PCa tissues are presented in Fig. 1a–d. Thirty-five tumor samples (52.24%) showed high MRPL23 expression, while 32 (47.76%) exhibited low expression.

MRPL23 expression was significantly higher in PCa tissues compared with normal tissue (*P* < .0001; Fig. 1e). Moreover, expression levels were also increased in lymph node metastases compared with primary tumors (*P* < .001, Fig. 1e) and significantly elevated in metastases compared with normal controls (*P* < .001, Fig. 1e). Consistent with these findings, a paired comparison of matched primary PCa tissues and their corresponding lymph node metastases demonstrated higher MRPL23 expression in metastases (*P* = .0128, Supplementary Figure S1). MRPL23 expression status was not significantly associated with clinicopathological features in our cohort (Table 1).

**Table 1.** Association of MRPL23 with clinicopathological features in our cohort of PCa patients.

Variables	*n* = 67	MRPL23		*P* value
		HIGH	LOW	
		*n* = 35	*n* = 32	
Age (years)				
<65	32 (47.76)	19 (59.38)	13 (40.63)	.33
>65	35 (52.24)	16 (45.71)	19 (54.29)	
Gleason score				
GS 6	3 (4.48)	2 (66.67)	1 (33.33)	.68
GS 7	35 (52.24)	16 (45.71)	19 (54.29)	
GS 8	11 (16.42)	7 (63.64)	4 (36.36)	
GS 9	18 (26.87)	10 (55.56)	8 (44.44)	
Grade group				
Group 1	3 (4.48)	2 (66.67)	1 (33.33)	.83
Group 2	11 (16.42)	5 (45.45)	6 (54.55)	
Group 3	24 (35.82)	11 (45.83)	13 (54.17)	
Group 4	11 (16.42)	7 (63.64)	4 (36.36)	
Group 5	18 (26.87)	10 (55.56)	8 (44.44)	
pT status				
pT2	9 (13.43)	4 (44.44)	5 (55.56)	.73
pT3–pT4	58 (86.57)	31 (53.45)	27 (46.55)	
pN status				
pN0	44 (65.67)	20 (45.45)	24 (54.55)	.20
pN1	23 (34.33)	15 (65.22)	8 (34.78)	
PSA				
<10 ng/mL	27(40.30)	13 (48.15)	14 (51.85)	.63
>10 ng/mL	40(59.70)	22 (55.00)	18 (45.00)	

### 3.2. The association between MRPL23 expression and patient survival

Kaplan–Meier survival analysis revealed that patients with high MRPL23 expression had significantly shorter OS rates compared with those with low expression (median OS: 1773 days versus not reached, *P* = .003; Fig. 1f). Univariate Cox regression analysis identified high MRPL23 expression as a significant predictor of poor OS (HR 3.43, 95% Cl 1.44–8.19, *P* = .005; Table 2). In the multivariable Cox proportional hazards model, high MRPL23 expression remained an independent prognostic factor (HR 3.99, 95% CI 1.63–9.77, *P* = .002; Table 2).

**Table 2.** Univariate and multivariate Cox proportional hazards regression analyses of prognostic factors in the institutional cohort.

Variable	Univariable analysis				Multivariable analysis			
	HR	95% CI		*P* value	HR	95% CI		*P* value
		L	U			L	U	
MRPL23 (present versus absent)	3.43	1.44	8.19	.01	3.99	1.63	9.77	<.01
Age (≥69 versus <69)	1.66	0.77	3.60	.20	2.20	0.96	5.02	.06
Gleason group (5 versus 1–4)	1.78	0.81	3.93	.15	1.55	0.69	3.48	.29
pT (pT3–pT4 versus pT2)	1.25	0.37	4.19	.72	—	—	—	—
pN(pN1versus pN0)	1.35	0.61	2.98	.46	—	—	—	—
AJCC stage (IV versus II–III)	1.35	0.61	2.98	.46	—	—	—	—
PSA (**>**10 versus <10)	1.38	0.64	3.00	.41	1.57	0.71	3.44	.26

### 3.3. In silico analysis of the TCGA PRAD cohort

**Figure 2.**
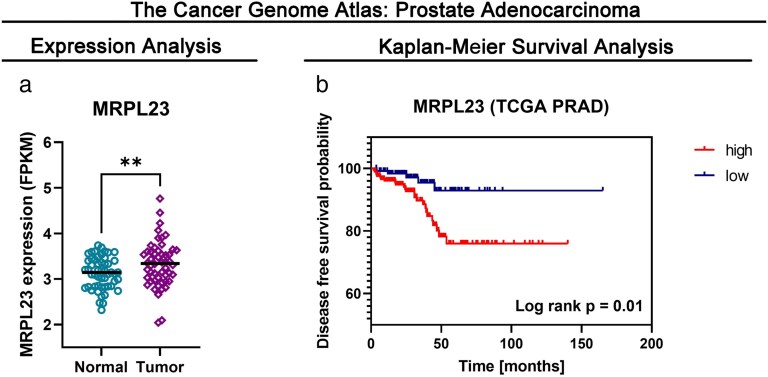
MRPL23 expression and prognostic value in the TCGA PRAD cohort. (a) MRPL23 expression in PRAD compared with normal tissues. (b) Kaplan–Meier analysis of DFS according to MRPL23 expression status

Normal tissue expression data were also extracted from the TCGA PRAD cohort. Patients characteristics are summarized in Supplementary Table S3. The mean MRPL23 expression was significantly higher in prostate adenocarcinomas than in normal tissues (FPKM 3.33 versus 3.13; *P* = .01; Fig. 2a).

We observed no correlation between MRPL23 expression and clinicopathological features of prostate cancer, such as lymph node invasion status, tumor stage, and patients age or race. There was also no association between adjuvant radiotherapy and MRPL23 expression (*P* > .05). There were also no correlations between progression-free survival or overall survival and MRPL23 status. However, the Kaplan–Meyer estimator showed that 10 years after diagnosis patients with low MRPL23 tumors had significantly higher disease-free rates than those with high MRPL23 tumors (92.92% versus 75.98%; log-rank *P* = .01; Fig. 2b).

Next, we employed the Cox proportional model to explore the prognostic role of MRPL23 in the TCGA PRAD cohort. In a univariate Cox regression model, radiotherapy, T4 tumor stage, and high MRPL23 expression were associated with disease-free survival (DFS). In a multivariate model, all factors retained significance as independent predictors of shorter DFS (Table 3).

**Table 3.** Univariate and multivariate cox proportional hazards regression analyses of prognostic factors in TCGA PRAD cohort.

Variable	Univariable analysis				Multivariable analysis			
	HR	95% CI		*P* value	HR	95% CI		*P* value
		L	U			L	U	
MRPL23 (low versus high)	3.27	1.25	8.54	.016	2.83	1.08	7.45	.03
Age (per year)	1.04	0.986	1.1	.15	—	—	—	—
Race	N/A	N/A	N/A	.99	—	—	—	—
Lymph node status (N0 versus N1)	1.88	0.72	4.95	.2	—	—	—	—
Tumor stage								
T2 versus T3	5.28	1.83	15.21	.55	4.38	1.49	12.84	.61
T2 versus T4	14.79	2.66	81.05	.015	11.66	2.09	65.1	.14
RTX (No versus Yes)	3.33	1.48	7.49	.004	2.36	1.03	5.44	.04

**Figure 3.**
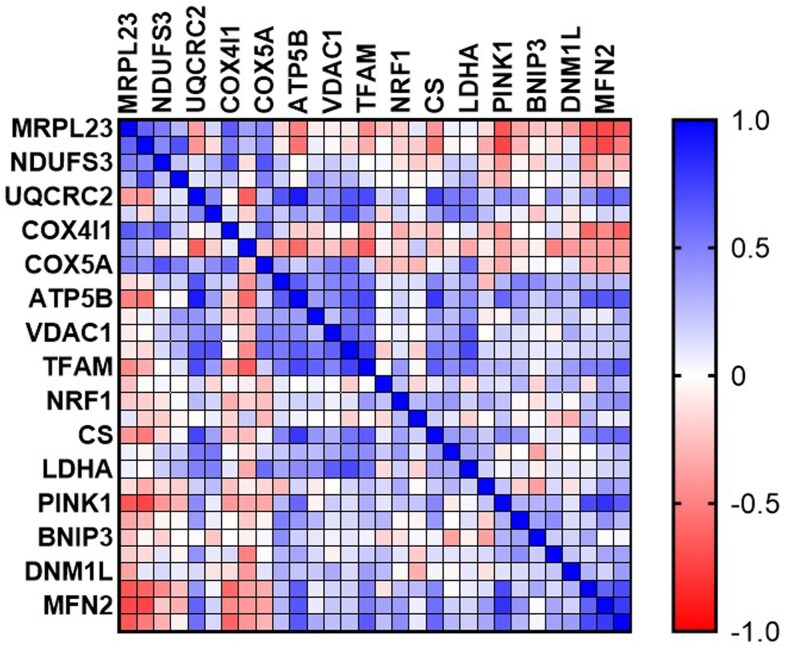
Correlation of MRPL23 expression with mitochondrial proteins in prostate adenocarcinoma (TCGA PRAD cohort)

Finally, we analyzed the correlation between MRPL23 expression and other mitochondrial proteins. The strongest correlations were noted between MRPL23 and NDUFS3 (*k* = 0.67), NDUFB8 (*k* = 0.65), and COX4I1 (*k* = 0.63). All results are presented in Fig. 3.

## 4. Discussion

In recent years, MRPL23 overexpression has emerged as a potential driver of progression in various cancer types [11, 15]. Research has explored its role not only in tumor development but also as a marker for risk stratification. However, a significant gap remains in our understanding of the role of MRPL23 expression in prostate cancer. Our study is the first to evaluate MRPL23 expression patterns in PCa, demonstrating that it is overexpressed in prostate cancer tissues compared with normal adjacent tissue, with the highest levels observed in lymph node metastases (Fig. 1e). Furthermore, our analysis revealed that high MRPL23 expression is an independent prognostic factor associated with shorter overall survival in PCa patients. These findings underscore the potential clinical relevance of MRPL23 as a biomarker of tumor aggressiveness. Importantly, complementary in silico analysis of the TCGA PRAD cohort confirmed elevated MRPL23 mRNA expression in prostate adenocarcinomas compared with normal tissues, although no significant associations with overall survival were found. Instead, high MRPL23 expression correlated with worse DFS, remaining an independent predictor in multivariate Cox regression alongside T4 stage and adjuvant radiotherapy. Together, these results suggest that MRPL23 influences disease recurrence and progression rather than overall mortality, highlighting its possible role in metastatic dissemination and therapy resistance.

To date, the mechanistic role of MRPL23 in oncogenesis remains poorly understood. In our previous study, we showed that clear cell renal cell carcinoma patients had lower MRPL23 expression levels than healthy controls. However, analysis of TCGA data revealed that mRNA expression levels were higher in tumor samples, suggesting that co-dependence and interplay between multiple signaling pathways are crucial for the crosstalk between the immune system and the tumor microenvironment [15].

While the role of MRPL23 in cancer biology is still under investigation, its antisense transcript, MRPL23 Antisense RNA 1 (MRPL23-AS1), has undergone more extensive studies. While both are located on chromosome 11p15.5, they have different biological functions: MRPL23 encodes a mitochondrial protein involved in translation and metabolism, whereas MRPL23-AS1 is a long non-coding RNA (lncRNA) with potential regulatory activity. Their close genomic proximity suggests possible coordinated roles in tumor progression [24]. For instance, high MRPL23-AS1 expression was identified as an independent prognostic factor for poorer survival in adenoid cystic carcinoma [25]. Zhang *et al*. also demonstrated similar effects of MRPL23-AS1 overexpression on OS in *in vitro* and *in vivo* osteosarcoma models, which may be attributed to increased viability and invasiveness of cancer cells [26] Researchers have also reported that MRPL23-AS1 predominantly localizes to the cytoplasm of osteosarcoma cells, where it binds to miR-30b, leading to increased MYH9 levels and activation of the Wnt/β-catenin signaling pathway, a well-known driver of tumor progression. In contrast, while the MRPL23 protein is predominantly localized to the cytoplasm and mitochondria, we did not investigate the molecular mechanisms underlying its role in tumorigenesis. Nevertheless, recent evidence suggests that MRPL23 may contribute to cancer development through multiple pathways, potentially influencing the clinicopathological characteristics of prostate cancer [6, 27, 28].

To gain further insight into the biological role of MRPL23, we analyzed its correlations with mitochondrial genes in the TCGA PRAD dataset. MRPL23 expression showed strong positive associations with OXPHOS components, including NDUFB8 and NDUFS3 (complex I) and COX4I1 (complex IV), while it correlated negatively with regulators of mitochondrial dynamics such as PINK1, MFN1, and MFN2. These findings suggest that MRPL23 upregulation may promote enhanced mitochondrial translation and OXPHOS activity, while being associated with impaired mitophagy and mitochondrial fusion. Such alterations in mitochondrial homeostasis could facilitate metabolic adaptation and contribute to prostate cancer progression.

Further insights into the tumor biology of MRPL23 come from *in vivo* studies focused on metastatic mechanisms. For instance, Soroosh *et al*. reported elevated MRPL23-AS1 expression in colorectal cancer samples with liver metastases, while no such increase was observed under *in vitro* conditions, suggesting that certain pro-metastatic effects may be context-dependent and mediated by factors present only in the tumor microenvironment [29]. Similarly, Chen *et al*. demonstrated that MRPL23-AS1 overexpression enhanced metastatic potential in adenoid cystic carcinoma cells, alongside altered expression of angiogenesis- and EMT-related markers, including E-cadherin, N-cadherin, and VEGF [30]. These findings are consistent with our observation of the highest MRPL23 protein expression levels in lymph node metastases, supporting the hypothesis that MRPL23 may actively contribute to metastatic progression in prostate cancer.

In addition to transcriptional activity, genomic variation at the MRPL23 locus may influence cancer susceptibility. Tan *et al*. identified germline polymorphisms (rs2839698 and rs3024270) associated with decreased MRPL23-AS1 expression and increased risk of hepatoblastoma [29]. Meanwhile, a study by Yuan *et al*. examined the rs2839701 variant, which was shown to reduce transcriptional activity and was associated with lower MRPL23-AS1 expression in oral squamous cell carcinoma (OSCC) [31]. However, the latter analysis was based on GTEx-derived data and lacked validation in OSCC tumor tissue. Notably, both studies focused on predisposition rather than disease progression, and did not account for microenvironmental or immune-related factors that might modulate MRPL23-AS1 expression in fully developed tumors.

Recognizing certain limitations of our analysis, we acknowledge that the study group was relatively small, which may affect the reliability of statistical conclusions. To obtain results with greater statistical power, further research should be conducted on a larger patient cohort. Moreover, future studies should integrate transcriptomic and proteomic data, ideally in a prospective setting, to clarify the observed discrepancies between mRNA and protein levels of MRPL23. Such combined approaches could help determine whether post-transcriptional mechanisms—such as altered mRNA stability, translational efficiency, or mitochondrial import—or technical and cohort-specific factors (e.g. batch effects in TCGA or tissue heterogeneity) underlie the inconsistent findings [32, 33]. Addressing these aspects will be essential to validate MRPL23 as a robust prognostic biomarker and to better understand its mechanistic role in PCa progression. Nonetheless, the consistency of our findings with the existing literature indicates that the role of MRPL23 may have clinically relevant functions, potentially informing the development of future therapeutic strategies. One promising direction is targeted therapy, where identifying MRPL23 as a factor contributing to cancer progression and metastasis may open up new perspectives for intervention.

In our cohort, MRPL23 expression was associated with prostate cancer progression and carried clear prognostic value. Elevated expression was associated with poorer clinical outcomes and decreased overall survival, suggesting that MRPL23 could serve as a biomarker for tumor aggressiveness and a potential therapeutic target.

## Supplementary Material

bgaf078_Supplementary_Data

## Data Availability

The datasets generated and analyzed during the current study can be obtained from the corresponding author upon reasonable request.

## Supplementary material

Supplementary material will be available at the *Carcinogenesis* journal website upon publication.

## References

[1] Khan MM, Sharma V, Serajuddin M. Emerging role of miRNA in prostate cancer: a future era of diagnostics and therapeutics. Gene 2023;888:147761. 10.1016/j.gene.2023.147761 37666374

[2] Rawla P . Epidemiology of prostate cancer. World J Oncol 2019;10:63–89. 10.14740/wjon1191 31068988 PMC6497009

[3] Sung H, Ferlay J, Siegel RL et al Global cancer statistics 2020: GLOBOCAN estimates of incidence and mortality worldwide for 36 cancers in 185 countries. CA Cancer J Clin 2021;71:209–49. 10.3322/caac.21660 33538338

[4] Leslie SW, Soon-Sutton TL, Ranasinghe A et al Prostate cancer. In: Newman W (ed.) Family Medicine: Principles and Practice, 8th ed. Amsterdam: Elsevier, 2023, 1407–15.

[5] Zhong X, He Z, Fan Y et al Multi-omics analysis of MRPL-13 as a tumor-promoting marker from pan-cancer to lung adenocarcinoma. Aging (Albany NY) 2023;15:10640–80. 10.18632/aging.205104 37827692 PMC10599762

[6] Chen CW, Fu M, Du ZH et al Long noncoding RNA MRPL23-AS1 promotes adenoid cystic carcinoma lung metastasis. Cancer Res 2020;80:2273–85. 10.1158/0008-5472.CAN-19-0819 32098781

[7] Bacon JM, Taylor S, Wyatt M et al Mitochondrial ribosomal proteins in metastasis and their potential as prognostic markers in cancer. Cancer Metastasis Rev 2024;43:1119–35. 10.1007/s10555-024-10216-4 39354291 PMC11554709

[8] Hao C, Duan H, Li H et al Knockdown of MRPL42 suppresses glioma cell proliferation by inducing cell cycle arrest and apoptosis. Biosci Rep 2018;38:BSR20171665. 10.1042/BSR20171456 PMC5920136 29531015

[9] Zeng Y, Shi Y, Xu L et al Prognostic value and related regulatory networks of MRPL15 in non-small-cell lung cancer. Front Oncol 2021;11:1479. 10.3389/fonc.2021.614126 PMC8138120 34026630

[10] Huang G, Li H, Zhang H. Abnormal expression of mitochondrial ribosomal proteins and their encoding genes with cell apoptosis and diseases. Int J Mol Sci 2020;21:8471–19. 10.3390/ijms21228471 33238645 PMC7700125

[11] Huang YB, Xu SM, Li M et al Suppression of MRPL23 induces cellular senescence in hepatocellular carcinoma by targeting HMGB1. Discov Oncol 2025;16:1–12. 10.1007/s12672-024-01698-3 40490666 PMC12149086

[12] Zhu J, Wen N, Chen W et al Mitochondrial ribosomal proteins: potential targets for cancer prognosis and therapy. Front Oncol 2025;15:1586137. 10.3389/fonc.2025.1586137 40371222 PMC12074914

[13] Redza-Dutordoir M, DA A-B. Activation of apoptosis signalling pathways by reactive oxygen species. Biochim Biophys Acta Mol Cell Res 2016;1863:2977–92. 10.1016/j.bbamcr.2016.09.012 27646922

[14] Denko NC . Hypoxia, HIF1 and glucose metabolism in the solid tumour. Nat Rev Cancer 2008;8:705–13. 10.1038/nrc2468 19143055

[15] Podemska E, Borowczak J, Łukasik D et al High expression of MRPL23 is associated with poor survival in clear-cell renal cell carcinoma. Cancers (Basel) 2024;16:3909. 10.3390/cancers16233909 39682098 PMC11640366

[16] Akter R, Awais M, Boopathi V et al Inversion of the Warburg effect: unraveling the metabolic nexus between obesity and cancer. ACS Pharmacol Transl Sci 2024;7:560–9. 10.1021/acsptsci.3c00301 38481689 PMC10928896

[17] Hanahan D, Weinberg RA. Hallmarks of cancer: the next generation. Cell 2011;144:646–74. 10.1016/j.cell.2011.02.013 21376230

[18] Amin MB, Greene FL, Edge SB et al The Eighth Edition AJCC Cancer Staging Manual: continuing to build a bridge from a population-based to a more “personalized” approach to cancer staging. CA Cancer J Clin 2017;67:93–9. 10.3322/caac.21388 28094848

[19] Piątkowska D, Klimaszewska-Wiśniewska A, Kosińska A et al Ubiquitin B, ubiquitin C, and β-catenin as promising diagnostic and prognostic tools in prostate cancer. Cancers (Basel) 2024;16:902–12. 10.3390/cancers16050902 38473264 PMC10930646

[20] Ogłuszka M, Orzechowska M, Jędroszka D et al Evaluate cutpoints: adaptable continuous data distribution system for determining survival in Kaplan–Meier estimator. Comput Methods Programs Biomed 2019;177:133–9. 10.1016/j.cmpb.2019.05.023 31319941

[21] Cerami E, Gao J, Dogrusoz U et al The cBio cancer genomics portal: an open platform for exploring multidimensional cancer genomics data. Cancer Discov 2012;2:401–4. 10.1158/2159-8290.CD-12-0095 22588877 PMC3956037

[22] Goldman MJ, Craft B, Hastie M et al Visualizing and interpreting cancer genomics data via the Xena platform. Nat Biotechnol 2020;38:675–8. 10.1038/s41587-020-0546-8 32444850 PMC7386072

[23] Budczies J, Klauschen F, Sinn BV et al Cutoff finder: a comprehensive and straightforward web application enabling rapid biomarker cutoff optimization. PLoS One 2012;7:e51862. 10.1371/journal.pone.0051862 23251644 PMC3522617

[24] Knight SB, Crosbie PA, Balata H et al Progress and prospects of early detection in lung cancer. Open Biol 2017;7:170070. 10.1098/rsob.170070 28878044 PMC5627048

[25] Zhang H, Liu S, Tang L et al Long non-coding RNA MRPL23-AS1 promotes tumor progression and carcinogenesis in osteosarcoma by activating Wnt/β-catenin signaling via inhibiting microRNA miR-30b and upregulating MYH9. Bioengineered 2021;12:162–71. 10.1080/21655979.2020.1863014 33356805 PMC8806232

[26] Xu YH, Deng JL, Wang LP et al Identification of candidate genes associated with breast cancer prognosis. DNA Cell Biol 2020;39:1205–27. 10.1089/dna.2020.5482 32456464

[27] Takahashi K, Yan IK, Kogure T et al Extracellular vesicle-mediated transfer of long non-coding RNA ROR modulates chemosensitivity in human hepatocellular cancer. FEBS Open Bio 2014;4:458–67. 10.1016/j.fob.2014.04.007 PMC4050189 24918061

[28] Bai Y, Zhang X, Li H et al Long noncoding RNA EZR-AS1 promotes tumor growth and metastasis by modulating Wnt/β-catenin pathway in breast cancer. Exp Ther Med 2018;16:2235–42. 10.3892/etm.2018.6461 30186463 PMC6122301

[29] Tan T, Li J, Wen Y et al Association between lncRNA-H19 polymorphisms and hepatoblastoma risk in an ethnic Chinese population. J Cell Mol Med 2021;25:742–50. 10.1111/jcmm.16124 33236528 PMC7812267

[30] Yuan Z, Yu Y, Zhang B et al Genetic variants in lncRNA H19 are associated with the risk of oral squamous cell carcinoma in a Chinese population. Oncotarget 2018;9:23915–22. 10.18632/oncotarget.23673 29844862 PMC5963630

[31] Li X, Zhao L, Liu L et al Genetic variants in mitochondrial ribosomal protein genes are associated with cancer susceptibility: evidence from a multicenter case-control study. Cancer Genet 2022;268–269:15–22. 10.1016/j.cancergen.2022.04.003

[32] Mertins P, Mani DR, Ruggles KV et al Proteogenomics connects somatic mutations to signalling in breast cancer. Nature 2016;534:55–62. 10.1038/nature18003 27251275 PMC5102256

[33] Leek JT, Scharpf RB, Bravo HC et al Tackling the widespread and critical impact of batch effects in high-throughput data. Nat Rev Genet 2010;11:733–9. 10.1038/nrg2825 20838408 PMC3880143

