# Supplementary material for: Analysis of MRPL23 protein expression and its role in prostate cancer pathogenesis
Source: Carcinogenesis. 2025 Dec 20;46(4):bgaf078. doi: 10.1093/carcin/bgaf078 (PMC12716982; doi:10.1093/carcin/bgaf078)
Supplement: bgaf078_Supplementary_Data [file bgaf078_supplementary_data.zip › Supplementary Figure 1.docx]

**Supplementary Figure 1**. Paired analysis shows higher MRPL23 expression in lymph node metastases compared with matched primary prostate cancer tissues.
