# Supplementary material for: Analysis of MRPL23 protein expression and its role in prostate cancer pathogenesis
Source: Carcinogenesis. 2025 Dec 20;46(4):bgaf078. doi: 10.1093/carcin/bgaf078 (PMC12716982; doi:10.1093/carcin/bgaf078)
Supplement: bgaf078_Supplementary_Data [file bgaf078_supplementary_data.zip › Supplementary Tables.docx]

**Supplementary Tables**

**Supplementary Table 1.** A detailed clinical characterization of patients within the institutional cohort.

| **Variables** | **n=67** |
| --- | --- |
| **Age (years)** |  |
| **<65** | 32 (47.76) |
| **>65** | 35 (52.24) |
| **Gleason score** | |
| **GS 6** | 3 (4.48) |
| **GS 7** | 35 (52.24) |
| **GS 8** | 11 (16.42) |
| **GS 9** | 18 (26.87) |
| **Grade group** | |
| **Group 1** | 3 (4.48) |
| **Group 2** | 11 (16.42) |
| **Group 3** | 24 (35.82) |
| **Group 4** | 11 (16.42) |
| **Group 5** | 18 (26.87) |
| **pT status** | |
| **pT2** | 9 (13.43) |
| **pT3-pT4** | 58 (86.57) |
| **pN status** | |
| **pN0** | 44 (65.67) |
| **pN1** | 23 (34.33) |
|  |  |
| **PSA** |  |
| **<10 ng/ml** | 27(40.30) |
| **>10 ng/ml** | 40(59.70) |

**Supplementary Table 2.** Antibodies and staining conditions used for immunohistochemistry.

| **Antibody** | **Company** | **Antigen retrieval** | **Incubation time** | **Dilution** | **Detection Kit** | **Substrate** |
| --- | --- | --- | --- | --- | --- | --- |
| **MRPL23** | Sigma- Aldrich  (HPA050406) | pH=8.5 CC1  buffer; 64 min | 32 min | 1:100 | Ventana Optiview DAB IHC Detection Kit | DAB |

**Supplementary Table 3.** Basic patients characteristics of the TCGA PTAD cohort.

| Clinical data | | Prostate adenocarcinoma, n(%)* |
| --- | --- | --- |
| Cases |  | 493 |
| Matched normal samples |  | 52 |
| Median age (years) |  | 61 (IQR 56-66) |
| Sex | Male | 493 (100%) |
| Race | White | 147 (29.82%) |
|  | Black or African-American | 7 (1.4%) |
|  | Asian | 2 (0.4%) |
| Neoadjuvant therapy | Yes | 2 (0.4%) |
|  | No | 491 (99.6%) |
| Adjuvant radiotherapy | Yes | 59 (12%) |
|  | No | 388 (78.7%) |
| Tumor stage | T2 | 186 (37.7%) |
|  | T3 | 290 (58.8%) |
|  | T4 | 10 (2%) |
| Lymph nodes invasion | N0 | 342 (69.4%) |
|  | N1-N3 | 78 (15.8%) |
| Progression | No progression | 400 (81.1%) |
|  | Progression | 93 (18.9%) |
| Progression-free survival (median) | | 25.71 months (IQR 13.81 - 44.81) |
| Disease-free status | Disease-free | 303 (61.5%) |
|  | Disease recurrence | 30 (6.1%) |
| Disease-free time (median) | | 30.34 months (IQR19.36 -45.73) |
| Follow-up time (median) | | 30.38 months (IQR 17.23 – 48.13) |
| Survival status | Alive | 483 (98%) |
|  | Dead | 10 (2%) |

IQR – interquartile range
* The total number of cases does not always sum to 493 due to missing data in the TCGA database.
